# Incisional hernia after surgical correction of abdominal congenital anomalies in infants: a systematic review with meta-analysis

**DOI:** 10.1038/s41598-020-77976-1

**Published:** 2020-12-03

**Authors:** Laurens D. Eeftinck Schattenkerk, Gijsbert D. Musters, David J. Nijssen, Wouter J. de Jonge, Ralph de Vries, L. W. Ernest van Heurn, Joep PM. Derikx

**Affiliations:** 1 Department of Paediatric Surgery, Emma Children’s Hospital, Amsterdam University Medical Centre, University of Amsterdam and Vrije Universiteit Amsterdam, Meibergdreef 9, 1005 AZ Amsterdam, The Netherlands; 2 Tytgat Institute for Liver and Intestinal Research, Amsterdam University Medical Centre, University of Amsterdam, Amsterdam, The Netherlands; 3 Department of General, Visceral-, Thoracic and Vascular Surgery, University Hospital Bonn, Bonn, Germany; 4 Medical Library, Vrije Universiteit, Amsterdam, The Netherlands

**Keywords:** Diseases, Gastroenterology, Health care, Risk factors

## Abstract

Incisional hernia (IH) in children could result in life-threatening complications, including incarceration and bowel strangulation. The incidence and risk factors of IH in infants are scarcely reported. Since IH-correction may require extensive surgery and a long recovery program, identifying infants and birth defects at risk, may lead to a different approach during the primary surgery. Therefore, the aim of this review is to systematically review the available data on the incidence of IH following surgery for congenital anomalies in infants. All studies describing IH were considered eligible. PubMed and Embase were searched and risk of bias was assessed. Primary outcome was the incidence of IH, secondary outcomes were difference in IH occurrence between disease severity (complex vs simple) and closure method (SILO vs primary closure) in gastroschisis patients. A meta-analysis was performed to pool the reported incidences in total and per congenital anomaly separately. Subgroup analysis within gastroschisis articles was performed. The 50 included studies represent 3140 patients. The pooled proportion of IH was 0.03 (95% CI 0.02–0.05; I^2^ = 79%, *p* ≤ 0.01) all anomalies combined. Gastroschisis (GS) reported highest pooled proportion 0.10 (95% CI 0.06–0.17; n = 142/1273; I^2^ = 86%; *p* ≤ 0.01). SILO closure (OR 3.09) and simple gastroschisis, i.e. without additional anomalies, (OR 0.18) were of significant influence. This review reports the incidence of IH in infants with different congenital abdominal anomalies, of which gastroschisis reported the highest risk. In GS patients, complex GS and SILO closure are risk factors for IH development.

## Introduction

Incisional hernia (IH) is a dreaded complication after abdominal surgery in children. It may result in serious life-threatening complications, including incarceration and bowel strangulation^1,2^. In addition, it is is a burden on the quality of life and may affect the development of the child^3,4^. The incidence and risk factors of IH in neonates are scarcely reported and therefore surgeons often refer to studies done in the adult population. For obvious reasons though, taking measures to prevent smoking and obesity in the infantile cohort would not aid in lowering IH rate^5^. The studies that do report IH in the paediatric population reported an incidence between 0.7 and 3.2%^6–9^. During the last decades major advancements have been made in the postnatal care of neonates with congenital anomalies. Those developments, such as extracorporeal membrane oxygenation (ECMO) and total parenteral nutrition, have increased survival of this crucial period^10^. Because more neonates survive, there has been an increase in abdominal paediatric surgery procedures^10,11^. As the total numbers of abdominal procedures rises, an increase in total accounts of IH is also to be expected.

IH correction may require extensive surgery and a long recovery program. Identifying infants and birth defects at risk, may lead to a different approach during the primary surgery. Therefore, the aim of this review is to estimate the incidence of incisional hernia following surgery for congenital anomalies in infants (less than 2 years of age) with a systematic review.

## Methods

Studies were selected according to the criteria outlined below based on the PRISMA Guidelines^12^. Our protocol has been registered with the International Prospective Register of Systematic Reviews (PROSPERO) on 7 March 2019 (registration number: CRD42019119268).

### Participants

All studies reporting on IH after surgical correction of abdominal congenital anomalies in infants were considered for this review. Studies reporting on other complications, animal studies, in vitro studies, non-English, conference abstracts and studies with less than ten cases were excluded.

### Search strategy

The electronic databases of the National Institutes of Health PubMed and EMBASE were systematically searched in February 2020 using both simple search terms as well as hierarchical family forms (e.g. MESH). The clinical data expert (RV) aided in the formation of the search strategy. The search combined four groups of search terms and their equivalents: (1) terms related to the age at the moment of surgery (e.g. *neonate*); (2) terms related to the location of surgery (e.g. *abdominal surgery*); (3) terms related to congenital abdominal defects (e.g. *omphalocele*); (4) terms related to incisional hernia (e.g. *cicatricial hernia)*. Mesh and search terms used in PubMed are included in Appendix 1.

### Primary and secondary outcomes

The primary endpoint was the pooled percentage of IH. Secondary endpoint included the pooled percentage per congenital anomaly and identification of possible risk factors such as surgical procedure and disease severity by means .

A Forest plot, containing the estimated overall pooled proportion of IH and the corresponding 95% CI was created using Graphpad Prism version 8. In the Forest plot we also reported the pooled proportion and CI per anomaly if; (1) IH was reported in at least three studies for the anomaly OR (2) if the total number of patients all studies on an anomaly combined was ≥ 100 patients; (3) there was at least one event of IH present all studies on a specific anomaly combined. If these criteria were not met the anomaly was reported under the forest plot as residual. These residuals were still included in the analysis for overall pooled proportions.

For all studies with multiple arms, data of both trial-arms were combined or, if only one arm matched the inclusion criteria, the appropriate arm was used. Additional extracted parameters were: author, country of conduct, year of publication, journal, study design, duration of follow-up, duration of study, number of participants, type of congenital anomaly, subdivision within anomaly, time to IH, repeat surgery because of IH, history of stoma data was extracted on. Duration of follow up was described as median with range, mean with/without standard deviation or citated text following each article’s own description. Duration was either reported in month or years recalculated from the reported value if necessary.

For studies describing gastroschisis, type of closure (by SILO or primary) and severity of disease (complex or simple) was noted following definitions used by the author. Studies deemed gastroschisis complex when a patient had additional anomalies, such as intestinal atresia. If the patient only suffered from a gastroschisis, it was accepted as simple gastroschisis.

### Data extraction

Titles and abstracts were screened by two independent authors (LES, DN) using Rayyan. Rayyan is an online based software that facilitates blind collaboration among reviewers. Disagreements were resolved by discussion between the two review authors. A third specialist author (JD or GM) was consulted if consensus was not reached. Afterwards the full text of the remaining articles was read to determine eligibility for inclusion (LES, DN). If the full text was not found the authors were contacted. Of the included articles the reference list was cross-checked to find any additional articles.

### Validity and eligibility assessment

All included articles were assessed for the methodological quality and risk of bias using the Newcastle Ottawa quality assessment scale^13^. LES and DN did the assessment separately.

### Data synthesis

For each study, a weighted average of the logit proportions of IH was determined by the use of the generic inverse variance method. The logit proportions were back transformed to the summary estimate and 95% CIs were obtained in a summary proportion representing the pooled proportion of the IH. Heterogeneity was assessed using the I^2^ and χ^2^ statistics. Analysis was performed using R-studio version 3.6.1 (package “meta” (Schwarzer, 2007) and “metaprop” (Viachtbauer, 2010)). The random-effects model was used for interpretation. Heterogeneity was deemed significant if the pooled data’s *p* value was < 0.05 or χ^2^ statistics were ≥ 75. Heterogeneity was interpreted as small (I^2^ ≤ 0.25), medium (I^2^ = 0.25–0.50) or strong (I^2^ ≥ 0.50), according to Higgins^14^. The gastroschisis subgroup analysis were performed using review manager (version 5.3) to generate forest plots. In this part of the meta-analysis the odds ratio was calculated using Mantel–Haenszel statistics, using the random-effects model to interpret results.

## Results

### Study characteristics

**Figure 1:**
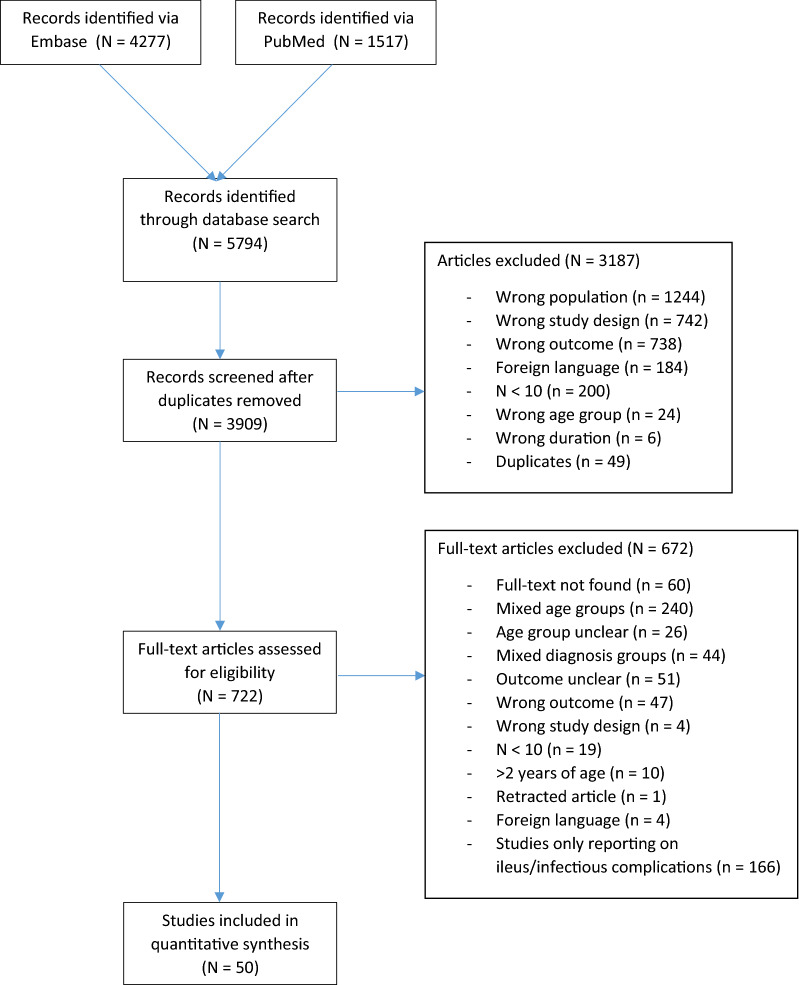
Flow diagram article selection.

During the search 5794 records were identified. Automated removal of duplicates left 3909 records for title and abstract screening. Of these 3909 records, 722 met the criteria for full text assessment. One article was included after screening references of included articles. Full text evaluation resulted in 50 studies, which were included for quantitative analysis (Fig. 1). These 50 studies represent 3140 patients (Table 1). Risk of bias was assessed and is shown in Table 2, most of the included studies reported fair quality on the NOS.

**Table 1:** Study characteristics.

Study (name, year)	Study design	Anomaly	IH/n	Follow-up	Time to hernia	Redo	Stoma	Type of surgery	Location of incision (n)
Dariel, 2015	Retrospective	Gastroschisis	6/41	3.1y (1–9)	165–1962d	5/6	No	NA	NA
Chiu, 2006	Retrospective	Gastroschisis	6/43	NR	NR	NR	No	NA	NA
Henrich, 2007*^1^	Prospective	Gastroschisis	3/22	6.3y (1–10)	NR	0/3	No	NA	NA
Payne, 2010	Case–control	Gastroschisis	2/127	3.3y (2.2)	NR	NR	No	NA	NA
Orion, 2011	Retrospective	Gastroschisis	14/44	11.4 m	“Prior to discharge”	4/14	No	NA	NA
Weil, 2011	Retrospective	Gastroschisis	43/190	NR	NR	NR	No	NA	NA
Friedmacher, 2014	Retrospective	Gastroschisis	3/108	15y (4–37)	NR	3/3	No	NA	NA
Tullie, 2016	Retrospective	Gastroschisis	10/39	53 m (10–101)	NR	NR	No	NA	NA
Dingemann, 2017	Multi-centre	Gastroschisis	2/39	“At least 1y”	4 months	2/2	No	NA	NA
Zmora, 2016	Retrospective	Gastroschisis	1/11	16 m	NR	NR	No	NA	NA
Mutanen, 2018*^1^	Multi-centre	Gastroschisis	3/34	Unclear	NR	3/3	No	NA	NA
Hawkins, 2019	Multi-centre	Gastroschisis	49/566	NR	NR	NR	No	NA	NA
Demirogullari, 2011	Retrospective	ARM	2/157	NR	NR	0/2	Yes	Laparoscopy	NA
de Vos, 2011	Retrospective	ARM	2/39	5.5y	NR	NR	Yes	Both	NA
Yang, 2014	Retrospective	ARM	0/20	1y	NA	NA	Yes	Laparotomy	Sub-umbilical (20)
Almosallam, 2016	Retrospective	ARM	0/104	NR	NA	NA	Yes	Laparotomy	NR
Diao, 2016	Retrospective	ARM	0/16	16 m (8–26)	NA	NA	No	Laparoscopy	NA
Ren, 2018	Retrospective	ARM	0/25	18 m	NA	NA	Yes	Laparoscopy	NA
Ren, 2019	Retrospective	ARM	0/48	59.4 m (13.7)	NA	NA	Yes	Laparoscopy	NA
Xiao, 2018	Retrospective	ARM	0/17	2.6y (2–4)	NA	NA	No	Laparoscopy	NA
Escobar, 2004	Retrospective	Duodenal obstruction	1/169	“Over 30ys”	10 months	1/1	No	Laparotomy	NR
Takahashi, 2010	Prospective	Duodenal obstruction	0/18	“Few months”	NA	NA	No	Laparotomy	Sub-umbilical (8) Transverse (10)
Kozlov, 2010	Retrospective	Duodenal obstruction	0/27	Unclear	NA	NA	No	Laparoscopy	NA
Ghaffarpour, 2013	Retrospective	Duodenal obstruction	0/28	“At least 1 m”	NA	NA	No	Laparotomy	Supra-umbilical
Jensen, 2013	Retrospective	Duodenal obstruction	2/66	NR	NR	NR	No	Both	Transverse (44)
Madadi-Sanjani, 2015	Retrospective	Biliary atresia	5/153	7.8 m (4.8–17.1)	NR	NR	No	Laparotomy	Transverse (153)
Bing, 2019	Prospective	Biliary atresia	1/25	25.4 m (6–59)	2 days	1/1	No	Laparoscopy	NA
Ramos-Gonzalez, 2019	Retrospective	Biliary atresia	2/81	5.7y (1–11.6)	NR	2/2	No	Laparotomy	Transverse (81)
Escobar, 2005	Retrospective	Hirschsprung	2/33	11y (9)	NR	2/2	No	Laparotomy	NR
Bianchi, 1998	Retrospective	Hirschsprung	1/13	“Range 7 m–13y”	NR	NR	No	Laparotomy	Oblique (13)
Teitelbaum, 1998	Retrospective	Hirschsprung	1/24	2.8y (1.7)	NR	1/1	No	Laparotomy	Oblique (24)
Santos, 1999	Retrospective	Hirschsprung	1/65	NR	NR	NR	NR	Laparotomy	NR
Sauer, 2005	Retrospective	Hirschsprung	1/24	7.3 m (9.7)	NR	1/1	No	Laparotomy	Umbilical (24)
Gao, 2019*^1^	Retrospective	Hirschsprung	1/35	NR	NR	1/1	NR	Laparotomy	NR
Jona, 2001	Retrospective	Hirschsprung	0/44	NR	NA	NA	No	Laparoscopy	NA
Ibrahim, 2012	Prospective	Diaphragmatic hernia	0/15	20 m	NA	NR	No	Laparoscopy	NA
Tyson, 2017	Retrospective	Diaphragmatic hernia	4/54	27 m (0.9–89)	NR	NR	No	Both	NR
de Bie, 2019	Multi-centre	Diaphragmatic hernia	0/62	“At least 1y”	NA	NR	No	Laparotomy	Sub-costal (62)
Dewberry, 2019	Retrospective	Diaphragmatic hernia	3/70	Unclear	NR	NR	No	NR	NR
Stollman, 2008	Retrospective	Small intestinal atresia	4/110	Unclear	NR	NR	No	Laparotomy	NR
Festen, 2002	Multi-centre	Small intestinal atresia	1/15	24 m (2–96)	9 months	NR	No	Laparoscopy	NA
Banieghbal, 2007	Prospective	Small intestinal atresia	0/16	6 m	NA	NA	No	Laparoscopy	NA
Li, 2012	Retrospective	Small intestinal atresia	0/35	“Range 2–27 m”	NA	NA	No	Laparoscopy	NA
Mutanen, 2018*^1^	Multi-centre	Small intestinal atresia	2/25	Unclear	NR	2/2	NR	Laparotomy	NR
Saxena, 2011	Prospective	Omphalocele	1/50	“3 years”	NR	1/1	No	NA	NA
Lee, 2006	Retrospective	Omphalocele	1/20	“Over 22ys”	NR	1/1	No	NA	NA
Henrich, 2007*^1^	Prospective	Omphalocele	3/15	6.3y (1–10)	NR	NR	No	NA	NA
Jiang, 2016	Retrospective	Omphalocele	4/24	NR	NR	NR	No	NA	NA
Zmora, 2016	Retrospective	Omphalocele	1/6	16 m	NR	NR	No	NA	NA
Michel, 2018	Retrospective	Omphalocele	0/16	11 m (1–48)	NA	NR	No	NA	NA
Diao, 2014	Retrospective	Choledochal cyst	0/27	24 m (1–50)	NA	NA	No	Laparoscopy	NA
van den Eijnden, 2017	Retrospective	Choledochal cyst	0/30	13.6y (0.8–26)	NA	NA	No	Both	NR
Önen, 2003	Combined	Meckel’s diverticula	0/34	NR	NA	NA	No	Laparotomy	NR
Gao, 2019*^1^	Retrospective	Meckel’s diverticula	0/12	NR	NA	NA	No	Laparotomy	NR

*^1^Henrich 2007, Mutanen 2018 and Gao 2019 each described 2 separate anomalies. *NA* not applicable, *NR* not reported.

**Table 2:** Assessment of risk of bias.

Author	Year	New Ottawa Scale (NOS)			
		Selection (0–4 asterisks)	Comparability (0–2 asterisks)	Outcome (0–3 asterisks)	Total (0–9)
Stollman	2008	***	*	***	7
Ibrahim	2012	***	–	**	5
Festen	2002	**	**	**	6
Escobar	2005	***	–	***	6
Dariel	2015	***	*	***	7
Demirogullari	2011	**	–	**	4
Bianchi	1998	***	–	***	6
Teitelbaum	1998	**	–	***	5
Santos	1999	***	–	***	6
Saxena	2001	***	*	***	7
Önen	2003	***	–	**	5
Escobar	2004	***	*	**	6
Sauer	2005	**	**	**	6
Chiu	2006	***	*	**	6
Lee	2006	**	*	***	6
Banieghbal	2007	**	–	***	5
Henrich	2007	***	–	*	4
Payne	2010	****	*	***	8
Takahashi	2010	***	*	**	6
de Vos	2011	***	–	***	6
Kozlov	2010	***	**	**	7
Orion	2011	***	–	**	5
Li	2012	***	–	**	5
Weil	2011	***	*	***	7
Ghaffarpour	2013	***	*	**	6
Jensen	2013	***	**	**	7
Diao	2014	**	*	***	6
Friedmacher	2014	***	**	***	8
Yang	2014	**	–	**	4
Madadi-Sanjani	2015	***	**	**	7
Almosallam	2016	***	–	**	5
Diao	2016	**	–	**	4
Jiang	2016	**	–	**	4
Tullie	2016	***	*	***	7
Dingemann	2017	***	–	***	7
Tyson	2017	***	*	**	6
van den Eijnden	2017	***	*	***	7
Zmora	2016	***	–	**	5
Michel	2018	***	*	**	6
Bing	2019	***	–	***	6
de Bie	2019	**	*	***	6
Dewberry	2019	**	**	**	6
Gao	2019	**	–	**	4
Jona	2001	**	–	**	4
Mutanen	2018	***	**	***	8
Ramos-Gonzalez	2019	***	*	***	7
Ren	2018	**	*	***	6
Ren	2019	**	*	***	6
Xiao	2018	**	*	***	6
Hawkins	2019	***	**	**	7

Six were prospective cohort studies, one combined a prospective-with a retrospective cohort, four were multicentre retrospective cohort studies, thirty-eight were retrospective cohort studies and one was a matched case–control study. None of the studies had a randomized design. Nineteen of the studies were conducted in North America, sixteen in Europe, eleven in Asia, three in Africa and one in the Middle-East. Follow up was at least half a year in 24 (48%) of all studies.

### Overall proportions

**Figure 2:**
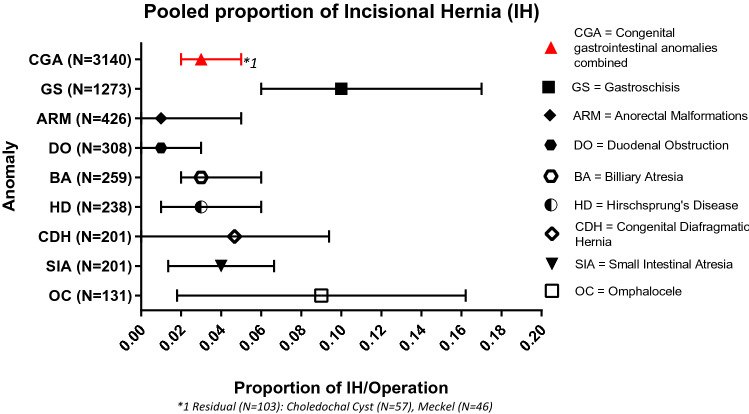
Pooled proportion of IH.

In 3140 patients 188 incisional hernias occurred^15–64^. None of the studies reported on the mode of diagnosis or the size of the defect. The pooled proportion of IH (Fig. 2) with all anomalies combined was 0.03 (95% CI 0.02–0.05; I^2^ = 79%, *p* ≤ 0.01). Separate pooled proportions were calculated for the following birth defects: Gastroschisis 0.10 (95% CI 0.06–0.17; n = 142/1273; I^2^ = 86%; *p* ≤ 0.01); Anorectal malformations 0.01 (95% CI 0.00–0.05; n = 4/426; I^2^ = 29%; *p* = 0.96); Duodenal obstruction 0.01 (95% CI 0.00–0.03; n = 3/308; I^2^ = 0%; *p* = 0.77); Biliary atresia 0.03 (95% CI 0.02–0.06; n = 8/259; I^2^ = 0%; *p* = 0.91); Hirschsprung’s disease 0.03 (95% CI 0.01–0.06; n = 7/238; I^2^ = 0%; *p* = 0.93); Congenital diaphragmatic hernia 0.03 (95% CI 0.01–0.10; n = 7/201; I^2^ = 34%; *p* = 0.91); Small intestinal atresia 0.03 (95% CI 0.02–0.07; n = 7/201; I^2^ = 0%; *p* = 0.91); Omphalocele 0.07 (95% CI 0.03–0.17; n = 10/131; I^2^ = 42%; *p* = 0.32). Choledochal cyst (N = 57) and Meckel (N = 46) are included in the overall proportion but were not reported separately as they did not meet the described criteria.

### Type of surgery

We calculated pooled proportions separately for all cases operated by laparoscopy and all cases operated by laparotomy as described in Table 1. For infants treated with by laparoscopy the pooled proportion was 0.01 (95% CI 0.01–0.03; n = 7/536; I^2^ = 0%; *p* = 0.00)^18,22,25,26,37,39,40,42,50,51,59,62^. For infants treated by laparotomy the pooled proportion was 0.02 (95% CI 0.01–0.04; n = 26/1098; I^2^ = 39%; *p* = 0.76)^15,17,21,22,28,29,32,33,37,43,45,46,49,52,53,55–57,59,63^. Of the studies that did report on the location of the incision, transverse incision was most reported. Therefore, no subgroup analysis was done based on the location of incision.

### History of stoma

All studies that reported on the history of stoma were articles describing anorectal malformations (ARM). Together, these studies contained four hernias. Two of which were not at the stoma site, but were reported as port-site hernia following laparoscopic assisted anorectoplasty^22^. The other two were seen at the site of stoma^23^. There were two articles describing ARM treatment without a stoma, no hernias occurred in these cohorts^26,62^.

### Gastroschisis

**Figure 3:**
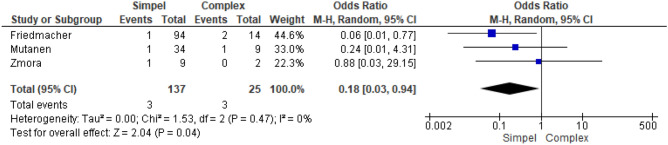
Forest plot simple vs complex GS.

Subgroup analysis within the GS studies showed a reduced risk of IH for simple gastroschisis compared to complex (Fig. 3); Odds ratio 0.18 (95% CI 0.03–0.94; *p* = 0.04; I^2^ = 0%).

**Figure 4:**
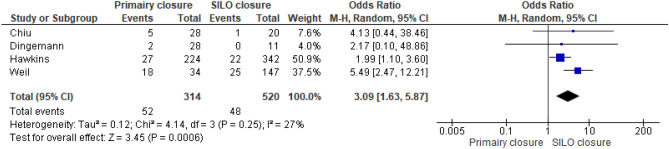
Forest plot SILO vs primary closure.

Also, an increased risk of IH was present when SILO closure was performed compared to primary closure (Fig. 4) with an odds ratio of 3.09 (95% CI 1.63–5.87; *p* ≤ 0.01; I^2^ = 27%).

## Discussion

This systematic review pooled the reported proportions on incisional hernia following abdominal surgery for congenital anomalies in infants. These proportions approximate the incidences of these complications. Therefore, our reported approximation of the incidence of IH was 3%. Within the different congenital anomalies gastroschisis patients are most at risk with an approximated incidence of 10%. In subgroup analysis complex gastroschisis (patients with GS and additional anomalies) and SILO closure were identified as risk factors for IH.

Studies reporting on laparoscopy and laparotomy seem to have similar incidences of IH although, due to the design of included studies, we were not able to compare studies. Still, this seems to be in line with previous cohort studies that focused on incisional hernia specifically in pediatric patients. These studies opted that the occurrence of IH seems to be more related to specific diagnosis instead of mode of surgery^6,7^. Within the included studies type of incision was only scarcely reported. Out of those that did, none reported the use of a midline incision. Transverse incision was the most reported type of incision. The use of this incision in infants provides a surgeon the best exposure to the abdominal cavity in neonates. The abdominal cavity of infants resembles a horizontal ellipsoid. With age and growth this ellipsoid changes into vertical position^65^. Thus, the younger the child the better visualization is obtained by using a transverse incision. Transverse incisions have been shown to have a lower incidence of IH compared to midline incision in adults^66,67^.

Previous studies suggested that the acquired newborn abdominal diseases necrotizing enterocolitis and pyloromyotomy are at higher risk for the development of IH^7,68^. Our review shows that the congenital abdominal wall defects gastroschisis and omphalocele should be added to this list.

Gastroschisis and omphalocele showed highest incidences of IH compared to the other congenital abdominal anomalies. Both anomalies are characterized by an abdominal wall defect with protruding viscera. In omphalocele, the viscera are covered with a membrane whilst in gastroschisis cases the viscera are not covered and thus more susceptible to infection. In order to surgically repair the defect, the viscera have to be reduced intra-abdominally. The primary goal of surgical repair is to close the defect as soon as possible, as to decrease the risk of infection, whilst simultaneously minimizing the risk of ischemic injury to the viscera due to increased intra-abdominal pressure (IAP)^69–72^. An increase of pressure between 0–15 mmHg is accepted because of the low risk of abdominal compartment syndrome^70^. However, even this increased outwards abdominal pressure during the wound healing process increases fascial tension, which could cause the increased incidence of IH^34^. Since 2004 an alternative method for a select group of patients named sutureless closure was proposed by methods of primary reduction and covering of the gastroschisis defect by watertight dressing and the umbilical cord^73^. This technique supposedly minimizes IAP and allows for spontaneous closure^74^. It is reported that this technique increases the risk of umbilical hernias compared to sutured closure. Still these hernias seem to resolve, not requiring redo surgery and sutureless closure seems to overall entail less complications^58,75,76^. This also might be the case for sutured closure. Out of six included studies that reported on redo surgery only 17 of the 31 hernias needed reoperation. It must be noted that most studies did not report on redo surgery.

Most gastroschisis cases are isolated conditions, but in 10% associated anomalies like intestinal atresias or necrotizing enterocolitis occur resulting in complex gastroschisis^72^. Our results show that simple gastroschisis has an odds-ratio of 0.18 to develop an IH compared to complex gastroschisis. In general, complex cases are associated with both longer parental nutrition and length of hospital stay^77^. This combination of impaired nutritional status and multiple anomalies could accumulate to a multifactorial cause of increased IH incidence.

We have shown that SILO closure increases the risk of IH compared to primary closure. SILO placement is mostly considered when the viscera cannot be reduced into the abdominal cavity because of bowel oedema. This oedema might result in higher IAP leading to an increase in the chances of IH. In a study comparing primary closure with SILO closure it was shown that the incidence of IH increased with longer duration of SILO reduction^34^.

Since the incidence in incisional hernia in the pediatric population is low, most cohort studies only describe limited numbers of patients with an incisional hernia. Still, in most of these studies thorough statistical analysis is done, often by chi-square or regression analysis. However, both analyses demand a sufficient amount of hernia cases ensuring the least expected count to be five for chi-squared statistics and a minimum of ten hernias per one controlling variable (events per variable (EPV)) for any form of regression analysis^78,79^. Not abiding these statistical rules can lead to inaccurate results causing conclusions to be, to some extent, unsupported or deceptive. Future research should ensure statistical power with a sufficiently large cohort. The incidence provided by this review can aid in power size calculations.

This review was limited because none of the included studies used a specific classification for incisional hernias or described the way they were diagnosed. Moreover, many studies did not report on duration of follow-up or had a follow up of less than a year, possibly resulting in missed incisional hernias. Also, different surgical approaches and disease severities were included. These differences dissolve when combining the study results into proportional meta-analysis, which could have influenced our results. The broad inclusion criteria, resulting in differences between compared studies, can partly explain the broad confidence intervals of gastroschisis and omphalocele. In adults, consensus has been reached that an incisional hernia should be classified by its location, length and width^80^. Whilst infantile incisional hernias might differ from adults, it seems advisable for prospective studies to include these characteristics as well as to describe the diagnostic modality as to aid in the generalizability of the interpretations. Using any form of diagnostic imaging will increase the total amount of detected IHs. Yet, this increase will be accompanied with disagreement between observers, as is the case in adults, making the IHs less generalizable which is why we suggest physical examination could be sufficient^81^. In our opinion, the clinical significance of IHs in infants undetectable by physical examination is doubtful. Smaller hernias have been reported to close without necessitating surgery, as is also the case in congenital umbilical hernias^7^. Since most IHs develop within a year after surgery it is desirable to plan an extra repeat visit at least a year after surgery as part of the research protocol^6^.

Another limitation of our study is that most included studies did not describe the incisional hernia cases in detail. Often, just the total number was noted without describing further case characteristics. This withheld us from thoroughly analyzing indications for redo-surgery or known risk factors such as having a history of stoma or surgical site infection. However, for gastroschisis specifically, though these finding are based on non-randomized retrospective cohort studies, our results suggest that SILO closure and complex cases are more at risk of IH. The high overall heterogeneity seems to be fairly explained by the spread in IH proportions within gastroschisis studies. This spread in its turn could be explained by differences in distribution of the described risk factors. Other anomalies showed moderate to low heterogeneity.

## Conclusion

This systematic review shows that the incidence of incisional hernia is 3% all studies on surgical correction of abdominal congenital anomalies in infants are combined. Overall the incidences seem comparable between studies reporting on laparoscopies and laparotomies. Gastroschisis patients are most at risk for developing IH with an approximated incidence of 10%. In subgroup analysis complex gastroschisis and SILO closure were identified as significant risk factors for IH.

## Supplementary information

Supplementary information.

## Data Availability

Data available on request.
